# Development of an Online, Evidence-Based Patient Information Portal for Congenital Heart Disease: A Pilot Study

**DOI:** 10.3389/fcvm.2017.00025

**Published:** 2017-05-01

**Authors:** Jonathan R. G. Etnel, Arie P. J. van Dijk, Jolanda Kluin, Robin A. Bertels, Elisabeth M. W. J. Utens, Eugene van Galen, Regina The, Ad J. J. C. Bogers, Johanna J. M. Takkenberg

**Affiliations:** ^1^Department of Cardiothoracic Surgery, Erasmus University Medical Center, Rotterdam, Netherlands; ^2^Department of Cardiology, Radboud University Medical Center, Nijmegen, Netherlands; ^3^Department of Cardiothoracic Surgery, Academic Medical Center, Amsterdam, Netherlands; ^4^Department of Cardiothoracic Surgery, Leiden University Medical Center, Leiden, Netherlands; ^5^Department of Pediatric Cardiology, Leiden University Medical Center, Leiden, Netherlands; ^6^Department of Child and Adolescent Psychiatry/Psychology, Erasmus University Medical Center – Sophia Children’s Hospital, Rotterdam, Netherlands; ^7^Research Institute of Child Development and Education, University of Amsterdam, Amsterdam, Netherlands; ^8^De Bascule, Academic Center for Child Psychiatry, Amsterdam, Netherlands; ^9^Patient Association ‘Patiëntenvereniging Aangeboren Hartafwijkingen’, Maarssen, Netherlands; ^10^ZorgKeuzeLab, Delft, Netherlands

**Keywords:** patient information, shared decision-making, congenital heart disease, patient education tools, patient information portal, multidisciplinary approach, International Patient Decision Aid Standards development process, Delphi technique

## Abstract

**Objectives:**

In response to an increased need for patient information on congenital heart disease in the Netherlands, we initiated a nationwide initiative to develop an online, evidence-based patient information portal, starting with a pilot project aimed at the subgroup of patients with congenital aortic and pulmonary valve disease.

**Methods and results:**

We developed an information portal that aims to (1) improve patient knowledge and involvement and to subsequently reduce anxiety and decisional conflict and improve mental quality of life and (2) to support physicians in informing and communicating with their patients. The information portal was developed according to the systematic International Patient Decision Aid Standards development process employing Delphi techniques by a multidisciplinary workgroup of pediatric and adult congenital cardiologists, a congenital cardiothoracic surgeon, a psychologist, an epidemiologist, a patient representative, and web and industrial design experts. First, patients and physicians were surveyed and interviewed to assess the current state of patient information and explore their preferences and needs to determine the focus for the development of the information portal. We found that patient knowledge and numeracy are limited, reliable information is scarce, physicians inform patients selectively and patient involvement is suboptimal, and there is a need for more reliable, tailored, and multi-faceted information. Based on the findings of these surveys and interviews, a patient-tailored information portal was designed that presents evidence-based disease- and age-specific medical and psychosocial information about diagnosis, treatment, prognosis, and impact on daily life in a manner that is comprehensible and digestible for patients and that meets the needs expressed by both patients and physicians. The effect of the website on patient outcome is currently being assessed in a multicenter stepped-wedge implementation trial.

**Conclusion:**

The present pilot project succeeded in developing an online, evidence-based information portal that is supported by both patients and physicians. The information portal will be further developed and expanded to include all other major forms of congenital heart disease, translations into other languages, and a public information portal to serve patients’ relatives and the general public at large.

## Introduction

Congenital heart disease is the most common congenital birth defect with an incidence of approximately 1% of all live births (1, 2). Due to major advances in the treatment of congenital heart disease over the past decades, approximately 90% of these patients now reach adulthood (3). This has, however, made congenital heart disease a chronic illness with, for example, an estimated 2.4 million people currently living with a congenital heart defect in the United States of America alone and an estimated 65,000 in the Netherlands.

The consequences of congenital heart disease for the individual patient are complex, time varying, and heavily dependent on the specific defect(s), individual patient-related factors, and treatment options and decisions. These consequences may have a significant impact on many facets of the patients’ lives, both clinical and personal. Therefore, informing patients and their relatives in a complete, objective, and understandable manner is essential in optimizing patient quality of life, lifestyle, health behavior, treatment adherence, and patient involvement in treatment decisions (4–16).

In response to an increased need for patient information in congenital heart disease in the Netherlands, we therefore initiated a nationwide initiative to improve patient information, starting with a pilot project aimed at a subgroup of congenital heart disease patients with aortic or pulmonary valve disease, including Tetralogy of Fallot (13–15, 17–21).

The objective of this pilot project was to develop an online information portal that aims to (1) improve patient knowledge and involvement and to subsequently reduce anxiety, depression, and decisional conflict and improve mental quality of life and (2) to support physicians in informing and communicating with their patients.

## Methods and Results

The present pilot study comprises a complete comprehensive development process for a target subgroup restricted to patients with congenital aortic and/or pulmonary valve disease and/or Tetralogy of Fallot as a proof of concept. The subsequent full-scale project will entail expansion to all other major forms of congenital heart disease, building on this proof of concept.

The focus of this pilot project was to develop a nationwide patient-tailored, evidence-based patient information tool to be incorporated into specialist congenital cardiac care developed by and for patients, caregivers, and physicians, based on both patient/caregiver and physician preferences.

First, we evaluated the current state of patient information in congenital heart disease in the Netherlands to determine key focus points for development. Next, we developed the information portal in a multidisciplinary national working group (Table 1) according to the systematic International Patient Decision Aid Standards (IPDAS) development process, employing Delphi techniques (22, 23). Finally, we designed and are currently conducting a stepped-wedge cluster randomized implementation trial. All three steps are described below.

**Table 1:** **Working group members**.

Role	Center	Appointed by
**Clinical**		
Patient representative^a^	–	Dutch Patient Association for Congenital Heart Disease
Pediatric cardiologist	LUMC, Leiden	Dutch Association for Pediatrics
Adult congenital cardiologist	Radboudumc, Nijmegen	Dutch Association for Cardiology
Congenital cardiac surgeon	AMC, Amsterdam	Dutch Association for Cardiothoracic Surgery
Clinical psychologist	Erasmus MC, Rotterdam	–
**Methodological**		
Epidemiologists	Erasmus MC, Rotterdam	Dutch Heart Foundation
Web and industrial design firm^b^	–	–

*^a^Chairman of the Dutch Patient Association for Congenital Heart Disease*.

*^b^Specialized in the development and implementation of patient information portals and decision aids*.

*–, not applicable; LUMC, Leiden University Medical Center; Radboudumc, Radboud University Medical Center; AMC, Academic Medical Center; Erasmus MC, Erasmus University Medical Center*.

## Evaluation of the Current State of Patient Information

The first crucial step in the development of the portal was a thorough evaluation of the current state of patient information and information needs in congenital heart disease in the Netherlands. The results of this phase would define the key focus points for the development of the information portal and, thus, represent the primary input for the next phase of the project.

We carried out this phase by conducting comprehensive surveys and interviews among patients (*N* = 63), caregivers of pediatric patients (*N* = 10), and physicians (*N* = 32). A detailed report of these surveys will be published separately, but the main findings included the following:

- *Patient/caregiver knowledge is limited*: although patients/caregivers *think* they are adequately informed, actual disease-specific knowledge was objectively sufficient in only half of the respondents, which is in line with previous findings (13, 14, 17–21).
- *Reliable information is scarce*: only 62% of patient/caregiver respondents agreed that reliable information was readily available to them. Subsequently, patients rely heavily on their physicians for information as evidenced by a mere 13% of patients citing sources other than their cardiologist or cardiac surgeon as one of their main sources of information.
- *Patient/caregiver numeracy is limited*: only 46% of respondents were able to successfully complete a 3-question basic numeracy test adapted from the Numeracy Scale (24, 25).
- *Patient/caregiver involvement is suboptimal*: both physicians and patients/caregivers agree that patients/caregivers are insufficiently involved. Physicians agree that most difficulty they experience in involving patients/caregivers is due to limited patient knowledge and comprehension.
- *Physicians inform patients/caregivers selectively*: as self-reported by physicians, the information they convey is mostly based on their own judgment of what is important and comprehensible to each patient/caregiver. This may not always correspond with what patients/caregivers themselves think is important.
- *Patient information preferences and needs*: in line with previous findings (26), the most important preferences and needs with regard to patient information expressed by *patients/caregivers* were as follows:
  ○ More (reliable) information on:
    ■ Implications for personal life (education, career, pregnancy, insurance, etc.)
    ■ Health behavior and lifestyle recommendations
    ■ Prognosis
    ■ Psychosocial aspects
    ■ Pros and cons of various treatment options
    ■ Recovery after surgery
  ○ Disease-specific information
  ○ Age-specific information
  ○ Non-contradictory information.
    Whereas *physicians* expressed a strong need for:
  ○ A single, trusted, evidence-based source of reliable patient information to which they can refer their patients
  ○ Tools to aid communication with patients/caregivers.

## Development of Information Portal

Based on the findings of the surveys and interviews and in response to the needs expressed by both patients and physicians therein, a first prototype of an information portal was drafted according to the IPDAS development process and employing Delphi techniques. This prototype was then internally reviewed and revised by all members of the working group in live meetings until a consensus was reached on all topics (Table 1) (alpha-testing). The resulting second prototype was then again extensively reviewed by independent adult patients (*n* = 2), caregivers of pediatric patients (*n* = 2), physicians (*n* = 6; two pediatric cardiologists, two adult congenital cardiologists and two congenital cardiac surgeons), and clinical psychologists (*n* = 2) from outside the working group, sampled from clinical practice (beta-testing). All testers were given specific instructions to focus on all aspects of the information portal, including information content, language, illustrations, design, and usability. Additionally, the patients/caregivers were also observed as they navigated the portal. The feedback from this beta-testing was the input for the final review and revision by the working group.

The product of this development process is a comprehensive patient information tool that corresponds with the preferences and needs expressed by patients and physicians and addresses the shortcomings identified in the surveys and interviews.

**Figure 1:**
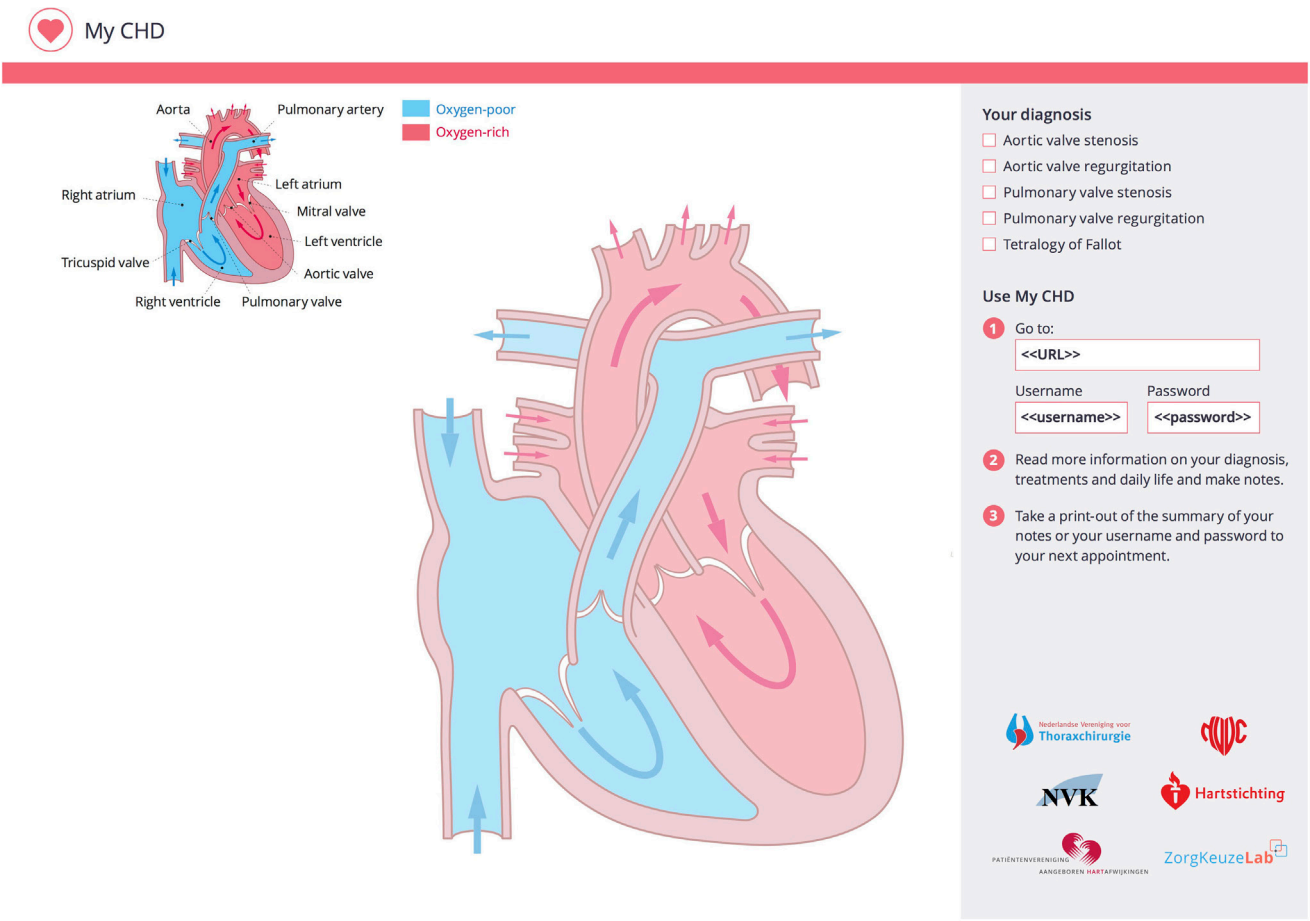
**Sketchpad**.

**Figure 2:**
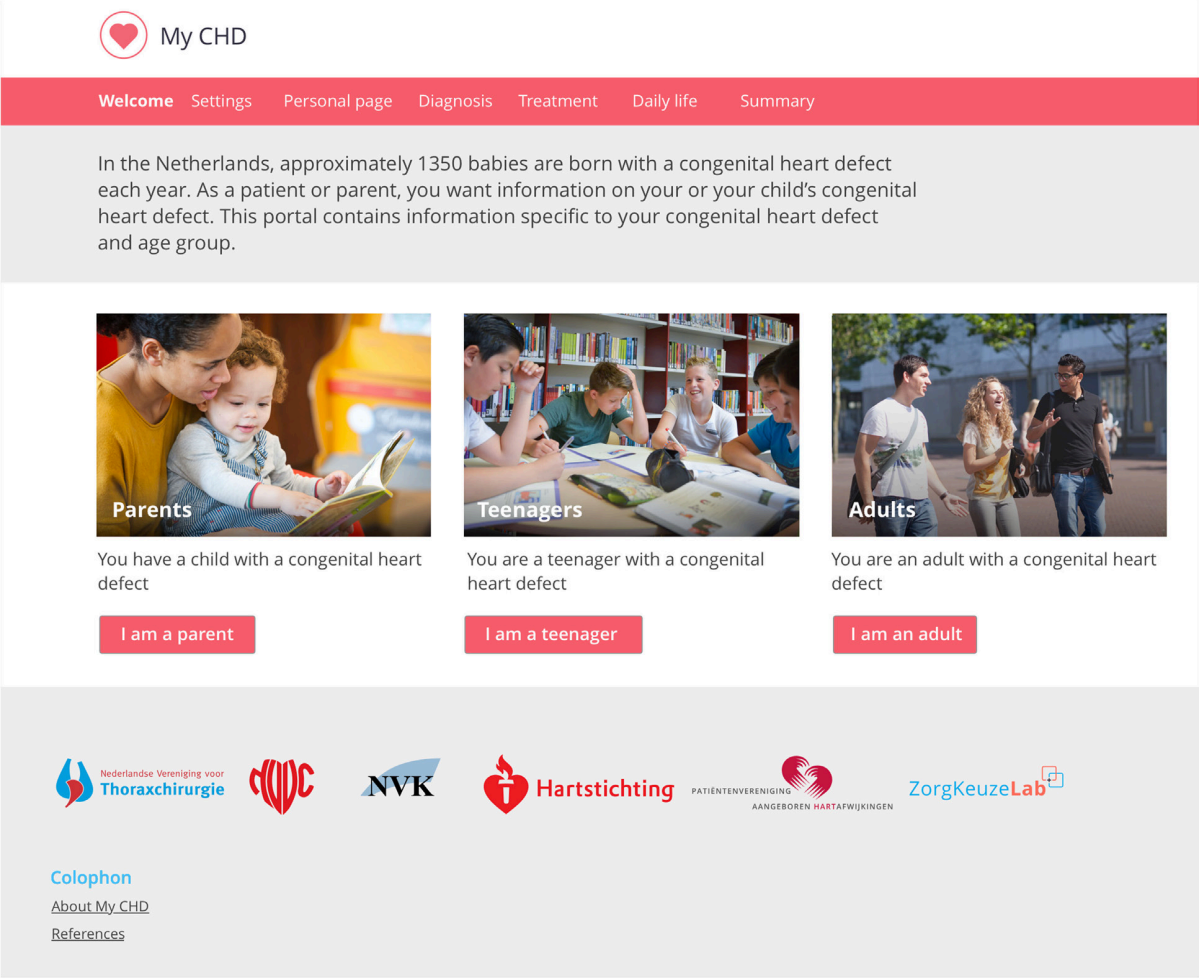
**Screenshot of the pilot online patient information portal**.

The implementation of the patient information portal in clinical care will take place as follows. Patients/caregivers that present to the cardiologist are invited to use the online information portal by the cardiologist who hands out a sketchpad during the consultation (Figure 1). This sketchpad offers a template for the cardiologist to provide a clear graphical representation of the patient’s heart defect as well as any other relevant notes for the patient/caregiver. On the sketchpad, the cardiologist also indicates which of the predefined diagnoses are applicable to the patient. The patients/caregivers can then take their sketch sheet home and review the cardiologist’s notes and drawings and visit the information portal using the link and personal private account details listed on the sketch sheet. When they do so, they enter a private information portal (Figure 2) with the following key characteristics.

### Disease- and Age-Specific Information

All information on the portal is compiled and presented specifically and separately for each congenital heart defect and target group (teenagers, adults, or parents/caregivers) with regard to both content and language.

Upon their first visit to the website, users are prompted to select their target group and diagnosis (two simple multiple choice prompts). Based on the combination of these inputs, a tailored personal subportal is custom built for each user. Their personal subportal contains only the information that is relevant to them. In case of multiple congenital heart defects, all relevant information is automatically combined into a single tailored subportal for that unique combination of inputs.

### Multi-Faceted Information Based on Patient/Caregiver Preferences

As patients/caregivers indicated a discrepancy between their own information needs and the information generally provided by physicians and other sources, the information provided by the portal is not based solely on what physicians think is important but rather represents both the clinical and the patient perspectives. Therefore, the information portal contains information on all aspects of disease that were found to be important to patients/caregivers and physicians in the surveys and interviews, such as diagnosis, treatment, prognosis, psychosocial aspects, and implications for daily life and future life planning.

### Format That Is Comprehensible and Digestible

To maximize digestibility and comprehensibility, the information is fragmented into various frequently asked questions that correspond with the topics that patients/caregivers and physicians indicated to be important in the surveys and interviews. Comprehension is further enhanced by the liberal use of custom illustrations, designed to the specifications of the multidisciplinary working group by a professional medical illustrator. Additionally, a professional medical text writer was contracted to optimize the linguistics of the textual content for each target group separately to maximize comprehensibility, digestibility and attractiveness for users of all ages and education levels. Furthermore, to address the limited numeracy among the target audience, all numerical risks on the information portal are supported by risk visualizations, such as icon arrays.

### Support Patient/Caregiver–Physician Communication

Patients/caregivers indicated that they are often unsure about which topics should be discussed with the physician. Throughout the information portal, we therefore provide numerous suggestions for important topics that should be discussed, as indicated by both physicians and patients/caregivers. Furthermore, there is a comment box on each page of the information portal in which patients/caregivers are encouraged to note any questions they may have about the information on that page. These questions are then saved in their personal account. Users can view, edit, and/or print a summary of their questions and optionally discuss this with their physicians.

Physicians and other involved health-care providers are also provided with their own personal accounts for the information portal, so that they can use the information portal to aid in explaining or illustrating disease-related information to patients/caregivers in the consulting room. Moreover, the sketchpad, as described above, is intended to further facilitate communication in the consulting room.

### Evidence-Based Information

All information on the information portal is based on international guidelines and peer-reviewed published evidence where possible. Furthermore, all four centers for congenital cardiac surgery in the Netherlands have combined their prospective databases of early outcome after all congenital cardiac surgery performed in these centers in the past 10 years to allow conveyance of reliable, nationwide data on risks and recovery after contemporary cardiac surgery to patients/caregivers.

## Implementation Trial

As the last phase of this pilot project, we are conducting a stepped-wedge cluster randomized (27, 28) implementation trial of the information portal in four large congenital cardiac centers in the Netherlands, which is ongoing as of writing.

The aim of this last phase of the pilot project is twofold:

- To gain insight into both the practical and cultural intricacies at each of the eight participating departments (departments of both adult and pediatric cardiology at each of the four participating centers) that need to be taken into account for effective implementation of the information portal and to subsequently develop individual implementation plans tailored to each of these departments and
- To evaluate the effect of the implementation of the information portal on patients and caregivers with regard to:
  ○ Disease-specific knowledge
  ○ Anxiety and depression
  ○ Mental quality of life
  ○ Patient/caregiver involvement and autonomy
  ○ Experiences with and views on patient information
  ○ Views on participation in decision-making
  ○ Decisional conflict.

Adult patients and caregivers of pediatric patients with congenital aortic and/or pulmonary valve disease and/or Tetralogy of Fallot that visit the outpatient clinic at one of the four participating centers are prospectively included. In total, at least 250 respondents will be included, 125 in the control group (no access to the information portal) and 125 in the intervention group (access to the information portal), all of whom will complete an online survey on the above topics 1 month after their visit to the outpatient clinic.

## Discussion and Further Development

The present pilot project succeeded in developing and implementing a nationwide online, evidence-based, disease- and age-specific information portal for (caregivers of) patients with congenital heart disease, based on extensive input from all parties involved in congenital cardiac care in the Netherlands and addressing both patient and physician needs. Our extensive and meticulous nationwide multidisciplinary development process ensures broad nationwide acceptance into clinical practice by both patients/caregivers and health-care providers.

In various disease states, more informed and activated patients have been previously found to be associated not only with improved quality of life, treatment adherence, health behavior, and clinical outcome but also with more efficient health-care utilization and lower health-care costs (4–16). The implementation trial, the final phase of the current pilot project, will shed light on the effect of the implementation of our pilot information portal on short-term psychosocial patient outcome. In the further development of the information portal, we will also focus specifically on clinical and long-term psychosocial effects as well as physician, implementation, and health-care service outcomes.

We are currently planning the further refinement and expansion of this information portal to all major forms of congenital heart disease, in which we aim to cover >90% of all cases of congenital heart disease. This full-scale project will build on all the knowledge, expertise, methods, framework, and infrastructure gained in the pilot project and will also be carried out in a multidisciplinary fashion. Additionally, focus groups with specific expertise will be employed when beneficial. This full-scale project will also include translations into other common languages, first and foremost English. We are also exploring innovative and interactive methods for improving patient participation, particularly in teenagers and adolescents.

Besides the further development of the current patient-tailored information portal, the full-scale project will also include the parallel development of a public information portal suited for broader use by patients and caregivers before a definitive diagnosis has been made, as well as their relatives and friends and the general public at large.

In the interest of sustainability, all relevant Dutch physician associations and patient associations have committed to a long-term partnership in this initiative. A multidisciplinary national working group in which each of these partners is represented will remain instated to oversee continuous review, updating, enhancement, and expansion of the information portal to ensure that we continue to provide up-to-date, evidence-based patient information of the highest standard.

Future partnerships and (conceptual) dissemination beyond the field of congenital heart disease and internationally may provide unique opportunities for further enhancing quality, expertise, and sustainability in this initiative.

## Ethics Statement

This study was approved by the institutional review board (MEC-2015-099), and all subjects gave written informed consent in accordance with the Declaration of Helsinki.

## Author Note

Presented at the Psychosocial Care from Fetus to Adult Meeting (16–18 March 2016, Rotterdam) of the Association for European Paediatric and Congenital Cardiology (AEPC) and at the 50th Annual Meeting (1–4 June 2016, Rome) of the AEPC.

## Author Contributions

Substantial contributions to the conception or design of the work; or the acquisition, analysis, or interpretation of data for the work; drafting the work or revising it critically for important intellectual content; final approval of the version to be published; and agreement to be accountable for all aspects of the work in ensuring that questions related to the accuracy or integrity of any part of the work are appropriately investigated and resolved: all authors (JE, AD, JK, RB, EU, EG, RT, AB, and JT).

## Conflict of Interest Statement

The authors declare that the research was conducted in the absence of any commercial or financial relationships that could be construed as a potential conflict of interest.
